# Fiber Structure—Property Relationships II: Macroscopic Deformations of Alkylene Sulfide Crosslinked Polycaprolactam Fibers

**DOI:** 10.6028/jres.066A.009

**Published:** 1962-02-01

**Authors:** Stephen D. Bruck

## Abstract

The introduction of alkylene sulfide crosslinks into oriented nylon-6 (polycaprolactam) homofiber is described. The resulting structural modifications are found to produce crimping and coiling in the dry and wet states of the fiber resembling the situation in wool. In a previous paper a similar phenomenon was reported for a disulfide crosslinked nylon-6 homofiber. The macroscopic deformations exhibited by the alkylene sulfide crosslinked fibers differ in. at least three respects from the disulfide crosslinked samples: (1) the crimp frequency is much higher and the crimp amplitude smaller, (2) in addition to crimping some helical coiling takes place even when the fiber is in the unswollen state, and (3) the extent of crimping and coiling is not decreased by gradual air-oxidation of some of the reactive groups. An explanation for this crimping and coiling previously advanced is corroborated by additional evidence provided by chemical analyses, and X-ray diffraction, birefringence, and swelling data.

## 1. Introduction

In previous communications [1, 2]^1^ it was shown that under suitable conditions, self-crimping and helical coiling can be achieved with round cross-section nylon-6 (polycaprolactam) homofiber by the introduction of disulfide crosslinkages. The resulting chemically modified fiber resembled wool in its bulking characteristics. The present paper deals with the introduction of alkylene sulfide crosslinks into oriented nylon-6 homofibers and describes some of the properties of the resulting structures.

## 2. Discussion

### 2.1. Introduction of Alkylene Sulfide Crosslinks

Although wool exhibits excellent bulking and other desirable properties, the possibility of improving some of its negative characteristics has challenged many workers. For example, the disulfide (cystine) crosslinks in wool are subject to degradation, reduction to the sulfhydryl groups (cysteine), and attack by microorganisms and enzymes. These problems prompted several early investigators [3, 4] to study the chemical modifications of the cystine crosslinks in wool.

In view of this work on wool, it seemed of interest to introduce longer, more flexible crosslinks of the alkylene sulfide types into nylon-6 fiber and to compare the properties of these structures with the previously reported disulfide crosslinked fibers. Discussion of the methods used to introduce the alkylene sulfide crosslinks is facilitated by reference to the reactions schemes at the top of page 78 (for details refer to Experimental Section).

The introduction of methoxymethyl groups (B) into nylon-6 polymer has already been described [1,5,6]. In order to convert (B) into the intermediate (C), the methylmethoxylated fiber was reacted with thioacetamide in the presence of methyl alcohol as swelling agent, and concentrated hydrochloric acid. (In previous work, thiourea was used, but it has been found that thioacetamide gives a similar product.) To facilitate the synthesis of a large number or alkylene sulfide crosslinks, it was necessary to inhibit the tendency of disulfide cross-links to form (E) by the oxidation of sulfhydryl groups (D). This was accomplished by reacting the intermediate (C) with an exces amount of potassium hydroxide to yield the potassium mercaptides (F) which are less sensitive to oxidation than sulfhydryls. The final steps involved the reaction of the potassium mercaptide groups with either methylene dihalide to yield the alkylene sulfide crosslinked product (G), henceforth referred to as type A, or alternatively, reacting the potassium mercaptide groups with ethylene dihalide to give the type B alkylene sulfide crosslinks (H). These last reactions are similar to the well-known Williamson synthesis of ethers from metallic alkoxides and alkyl halides [7].

Although the potassium mercaptide groups are less vulnerable to oxidation than the sulfhydryl groups, the final product undoubtedly contains some disulfide crosslinks and also some “blocked” (alkylated) groups of the type:

**Figure:**
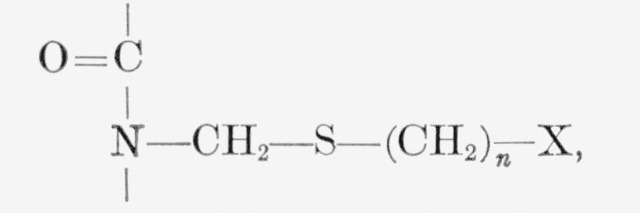


where, *n*= 1 or 2, and X = I or Br. The presence of these latter groups could be expected since, because of steric factors, not all potassium mercaptide groups can form crosslinks and therefore are subject only to the blocking reaction. No significant amounts of free potassium mercaptide or sulfhydryl groups were detected in the alkylene sulfide crosslinked fibers by a sensitive technique [8], specially devised for this purpose.

**Figure:**
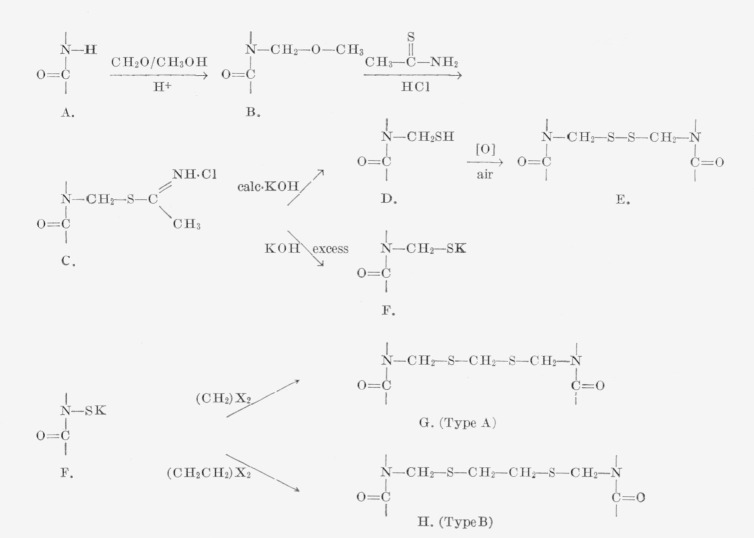


Where, X = I or Br.

The pertinent analytical data are summarized in table 1. The percent sulfur represents the combined quantities of alkylene sulfide and disulfide crosslinks, as well as the “blocked” (alkylated) sulfhydryl groups. The total sulfur content of the samples varied between 2.7 to 3.7 percent depending on the type of crosslinks and on the experimental conditions. The relative extent of crosslinking was also estimated by a photomicrographic swelling technique [9,10]. The symbol *q_m_* represents the ratio V/V_0_ of the volumes of the swollen to the unswollen structures at equilibrium [11]. The minimum observed *q_m_* values were between 5.1 and 5.8, indicating that a high density of alkylene sulfide crosslinks had been introduced into the fiber. Assuming that there were no side reactions, the total sulfur analyses indicates approximately 400 to 500 equivalents of crosslinks per 10^6^ g of polymer. Although these crosslinks were probably not uniformly distributed in the fiber, dyed cross sections indicated that, as in the case of the disulfide cross-links, the alkylene sulfide crosslinks extended throughout the entire structure and were not confined to the surface [12].

### 2.2. Macroscopic Deformations of Alkylene Sulfide Crosslinked Nylon-6 Fibers and Their Structural Significance

The alkylene sulfide crosslinked nylon-6 homofibers exhibited self-crimping and helical coiling of uneven distribution and dimensions in both dry and wet states similar to those already reported for disulfide crosslinked fibers. However, these macroscopic aeformations differed in at least three respects from those of the disulfide crosslinked samples: (1) the crimp frequency was much higher and the crimp-amplitude smaller, (2) in addition to crimping, some helical coiling occurred even when the fiber was in the unswollen state, and (3) the extent of crimping and coiling was not gradually decreased by air oxidation of unreacted sulfhydryl groups.

**Figure 1:**
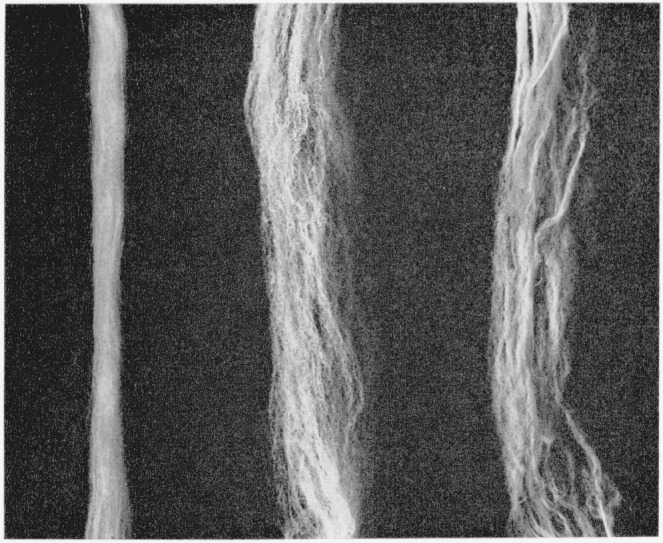
Crimping of alkylene sulfide crosslinked nylon-6 fibers. Left: unmodified, Middle: type A crosslinks; Right: type B crosslinks.

**Figure 2:**
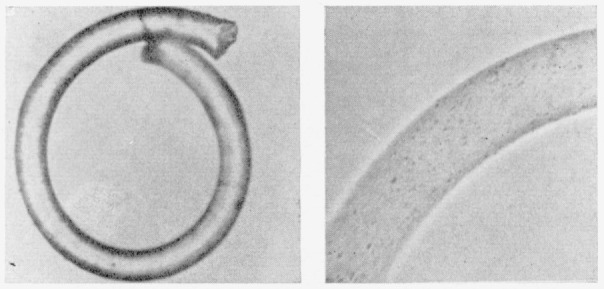
Helical coiling of type A alkylene sulfide crosslinked nylon-6 fiber. Left: A single helical coil of the unswollen (dry) fiber; Right: A portion of the helical coil swollen in *m*-cresol.

**Figure 3:**
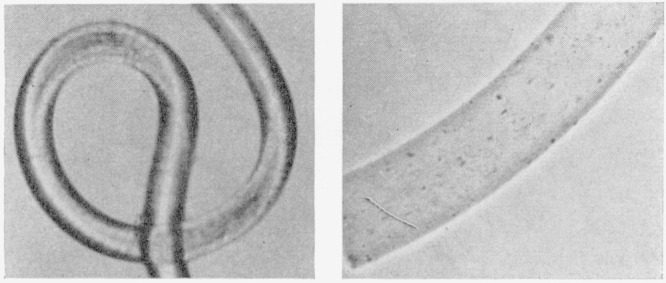
Helical coiling of type B alkylene sulfide crosslinked nylon-6 fiber. Left: A single helical coil of the unswollen (dry) fiber; Right: A portion of the helical coil swollen in *m*-cresol.

Figure 1 is a photograph of the unmodified (scoured) nylon-6 fiber, the type A crosslinked fiber and the type B crosslinked fiber. Figure 2 shows a photomicrograph of a single helical coil in the unswollen fiber (type A) and a phase-photomicrograph of a portion of the same coil while swollen in *m*-cresol. The diameter of the fiber before swelling was between 16 and 18 *μ* and the magnification in both photographs is the same. The small spots appearing in the swollen fiber are due to particles of TiO_2_ delustrant present in the fiber in small amounts. Figure 3 shows similar photographs of a fiber with type B crosslinks before and while swollen in *m*-cresol. The fiber diameter before swelling was between 20 and 23 *μ*, and the magnification in both photographs is the same. When either type A or B crosslinked fibers were swollen with *m*-cresol, additional helical coils were produced.

In the previous paper [1] a tentative hypothesis was advanced to explain the crimping and coiling tendency in terms of differential swelling and structural asymmetry resulting from the crosslinking process. Since then, additional data on the crystalline structure and internal orientation of crosslinked nylon-6 homofibers have been obtained, and the effect of alkylene sulfide crosslinks on fiber properties has been studied. These new data provide an increased understanding of the effects of crosslinking on fiber properties and will be presented as part of the following discussion.

From the chemical reaction series discussed above, it is noted that prior to the introduction of alkylene sulfide crosslinks, some disulfide crosslinks will have been formed due to partial oxidation of the mercaptide groups. These crosslinks exercise a local restraining effect upon the amorphous structure and some crimping results during the mild swelling that occurs at this stage. The next step actually involves two simultaneous reactions: (1) Further swelling of the fiber in a mixture of benzyl alcohol and ethyl alcohol, and (2) additional crosslinking by flexible alkylene sulfide groups. During this swelling process further internal plasticization of the fiber and partial disruption of the crystallites take place, thus permitting the penetration of the alkylene dihalide reagent. The crystallites present resist the deformation of the fiber, but as the smaller crystallites are disrupted, some of this resistance is removed so that the crimping becomes more pronounced and some coiling occurs. It is likely that the introduction of alkylene sulfide crosslinks when the fiber is in this deformed state tends to reinforce this deformation, and to accentuate the extent of crimping and coiling even in the unswollen state. If complete destruction of the crystallites is permitted, such as during swelling in *m*-cresol, the fiber will undergo helical coiling because all counterforce to deformation has now been removed. This latter effect is similar to that previously observed in the disulfide crosslinked system [1, 2].

The above macroscopic deformations are associated with changes in the fine structure of the fiber as evidenced by birefringence and X-ray analyses. Birefringence measurements were carried out on the uncrosslinked and crosslinked nylon-6 fibers using a slot compensator (sodium D light) with samples approximately 2 in. in length. Birefrengence= (*n*_1_−*n*_2_) = *R/t*, where *n*_1_ and *n*_2_ are refractive indices along and across the fiber axis, respectively, *R* is retardation in millimicrons (measured with the compensator), and *t* is thickness of the fiber in millimicrons (measured with a micrometer eyepiece). The birefringence values of the uncrosslinked (scoured) samples varied between +0.055 and + 0.060. On the other hand, the allcylene sulfide cross-linked fibers showed nonuniform birefringence values that ranged from + 0.019 to + 0.0048 for five measurements along approximately 2 in. length samples. The irregular decrease in birefringence indicates decreased and nonuniform orientations of the polymer chains in the crosslinked fiber. This observation is similar to that reported earlier in the disulfide crosslinked samples [1]. The decrease in birefringence is probably the result of the partial disruption of the crystallites and the plasticizing effect of the swelling agent, and is limited by the structural strain caused by the crosslinks.

**Figure 4:**
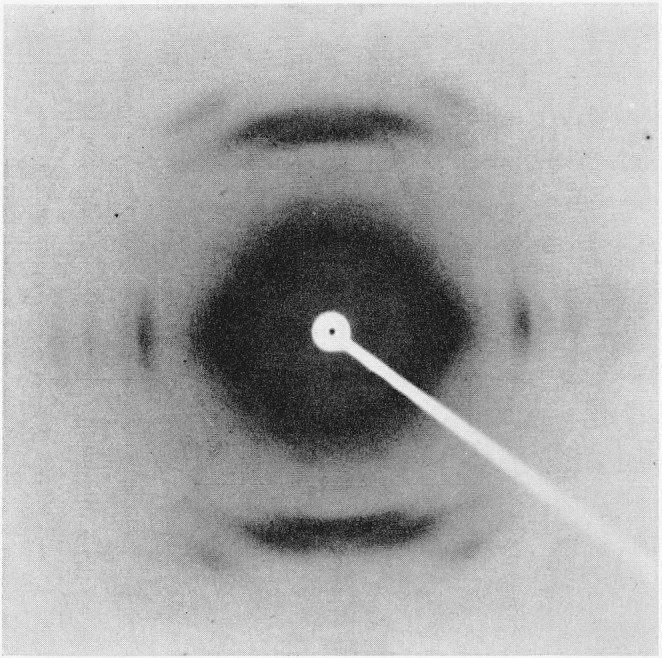
X-ray diffraction photograph of unmodified (scoured) nylon-6 fiber.

**Figure 5 f5-jresv66an1p77_a1b:**
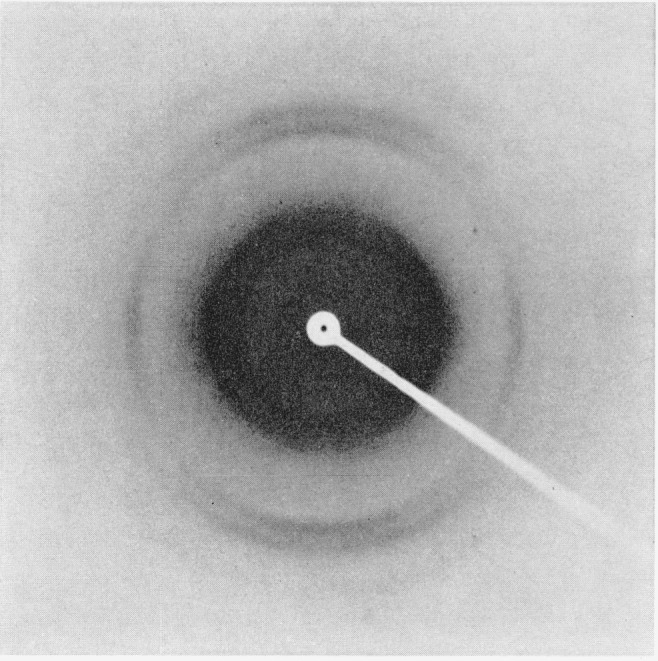
X-ray diffraction photograph of disulfide crosslinked nylon-6 fiber.

**Figure 6:**
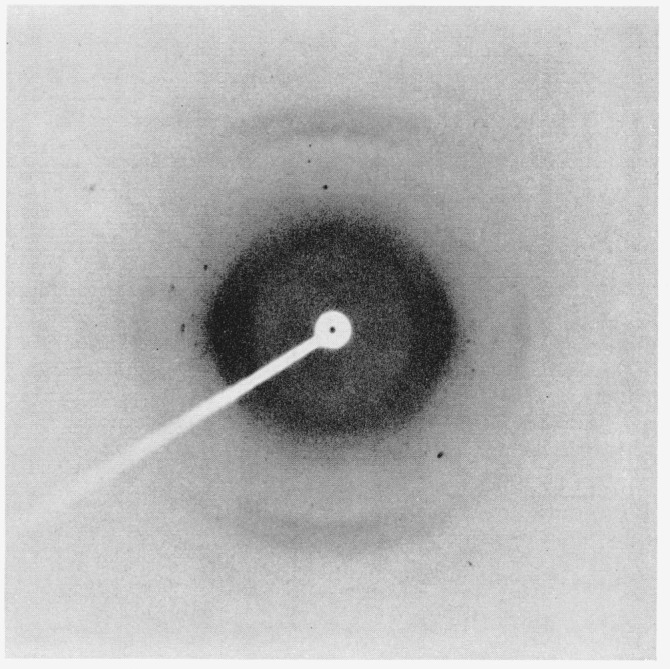
X-ray diffraction photograph of alkylene sulfide crosslinked (type A) nylon-6 fiber.

Additional evidence for important changes in the internal structure of crosslinked nylon-6 fibers is shown by X-ray analyses [13, 14, 15]. Figures 4 to 6 depict X-ray diffraction photographs of the uncrosslinked (scoured), disulfide crosslinked, and alkylene sulfide crosslinked fibers, respectively. [Fig f5-jresv66an1p77_a1b]

The unmodified nylon-6 (fig. 4) exhibits good orientation, whereas the alkylene sulfide crosslinked sample (fig. 6) shows appreciable orientation spread of up to around 20° from the ideal unit-cell alinement. The disulfide crosslinked sample (fig. 5) shows even less preferred orientation. The innermost of the two intense equatorial spots has been spread into a continuous Debye-Scherrer ring that shows only slightly greater intensity along the equatorial compared to that along the meridional radius.

It is not yet possible to differentiate between the relative effects of decreased orientation and decreased crystallite size on the observed macroscopic deformations exhibited by the disulfide and alkylene sulfide crosslinked samples. The partial disruption of the crystallites may be due to the combined effects of plasticization by the swelling agent and to internal strain caused by the crosslinks. The observed higher crimp frequency and partial helical coiling even in the unswollen (dry) state of the alkylene sulfide crosslinked fibers in comparison to the disulfide crosslinked samples may thus indeed be the result of the removal of some of the counterforce maintained by the crystallites, as already discussed above. (See note on p. 81.)

In contrast to the disulfide crosslinked nylon-6 fibers [1], the alkylene sulfide crosslinked samples showed no decrease in the extent of crimping and coiling after prolonged standing in air or when the fibers were treated with dilute solutions of an oxidizing agent, such as H_2_O_2_ This is probably due to the fact that in the case of the alkylene sulfide crosslinked fibers there were no significant amounts of free labile sulfhydryl groups, these having been “blocked” (alkylated) by either methylene or ethylene halides from further oxidation. Thu, the “built-in” differential swelling effect [1] was maintained. The “blocking” of the free sulfhydryl groups can also be achieved by treatment with AgNO_3_, thus forming the stable silver mercaptides, but this procedure causes blackening of the fibers due to reduction of some of the silver ions to free silver that cannot be readily removed.

The alkylene sulfide bonds are much less subject to chemical reductions, than fire the disulfide groups [16]. This property should make the former crosslinkages more desirable from the point of view of stability. It is possible that other properties, such as heat and hydrolytic stability, as well as stress-strain characteristics, will also show decisive advantages for the alkylene sulfide type bonds over the disulfide crosslinkages.

## 3. Experimental Details

All work was carried out with 7.8 Tex (60 denier)/32 filament round cross-section nylon-6 homofiber.

### 3.1. *N*-methoxymethylation (B)

This reaction was carried out according to a procedure reported earlier [1, 5, 6].

### 3.2. Preparation of the Mercaptides (F), Experiment No. 1, Table 1

A small skein (0.1–0.2 grams) of the *N*-methoxymethylated nylon-6 fiber was soaked for 12 hr at room temperature in a solution containing 37.5 g (0.5 moles) of thioacetamide and 700 ml of methyl alcohol. Next, 35 ml of conc. HOI (0.42 moles) was added and the fiber soaked for 1 hr at room temperature. After this period 60.0 g (1.06 moles) of KOH was added in 200 ml of methyl alcohol and the fiber was permitted to soak at room temperature for 1 hr. The fiber was then washed thoroughly twice with methyl alcohol, twice with distilled water, and again twice with methyl alcohol.

### 3.3. Introduction of Type A Alkylene Sulfide Crosslinkages (G), Experiment No. 1, Table 1

- The fiber from (2) was soaked for 5 hr at room temperature in a solution of 150 ml of ethyl alcohol, 150 ml of benzyl alcohol, and 29 ml (0.36 moles) of methylene diiodide. After this period the sample was thoroughly washed twice with ethyl alcohol, twice with distilled water, and twice with methyl alcohol and allowed to dry at room temperature. S= 3.7 percent.
- The procedure was the same as in (a), except that soaking time was 24 hr instead of 5 hr (Exp. No. 2, table 1). S= 3.6 percent.

### 3.4. Introduction of Type B Alkylene Sulfide Crosslinkages (H), Experiment Nos. 3 and 4, Table 1

The fiber from (2) was soaked for 24 hr at room temperature in a solution of 90 ml of ethyl alcohol, 90 ml of benzyl alcohol, and 90 ml (1.04 moles) of 1,2,-dibromoethane. After this period the sample was thoroughly washed twice with ethyl alcohol, twice with distilled water, and twice with methyl alcohol, and allowed to dry at room temperature. S= 2.9 percent.

### 3.5. Introduction of Disulfide Crosslinks (E), Experiment No. 5, Table 1

This reaction was carried out according to a procedure reported earlier [1]. S=2.7 percent.

## Figures and Tables

**Table 1:** Analytical data on crosslinked nylon-6 fibers

Experiment^1^	Total S	Predominant crosslink type^2^	Swelling ratio *q_m_*^3^	Macroscopic deformation	
				Dry or wet	*m*·cresol
					
	*wt %*				
1	3.7	A	8.0	Very highly crimped; some helices.	Helices.
2	3.6	A	5.1	do	Do.
3	2.9	B	6.2	do	Do.
4	2.9	B	6.8	do	Do.
5	2.7	O	5.8	Crimped	Do.

1 Refer to Experimental Section.

2
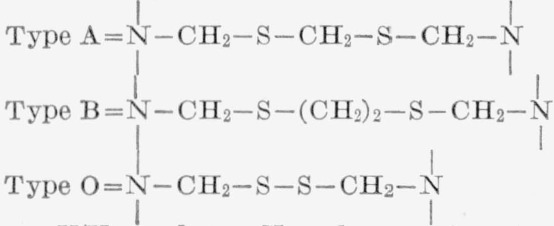

3 *q_m_*=V/V_0_, where V =volume of network at equilibrium swelling V_0_=volume of network before swelling. Swelling agent; *m*·cresol.

## Footnotes

1 Figures in brackets indicate the literature references at the end of this paper.
